# Electroanatomical properties and high-density mapping guided targeted isolation of superior vena cava

**DOI:** 10.1186/s12872-026-06215-8

**Published:** 2026-07-02

**Authors:** Vera Maslova, Hannah Budke, Adrian Zaman, Martina E. Spehlmann, Derk Frank, Evgeny Lian

**Affiliations:** 1 Department of Internal Medicine III, Cardiology and Angiology, University Hospital Schleswig-Holstein, Arnold-Heller Str. 3, Kiel, 24105 Germany; 2 German Centre for Cardiovascular Research (DZHK), Partner Site North, Kiel, Germany

**Keywords:** Atrial fibrillation, Non-pulmonary vein triggers, Superior vena cava

## Abstract

**Introduction:**

Superior vena cava isolation (SVCI) is associated with complications such as sinus node (SN) and phrenic nerve (PN) injury or SVC stenosis. Our study assessed the electrophysiological properties of the SVC and use of high-density mapping for targeted SVCI by ablating preferential conduction sites to avoid these complications.

**Methods:**

Eighty-three consecutive patients, undergoing HD mapping of the SVC during AF re-ablation procedures were prospectively included. Conduction block (CB) lines between the right atrium and SVC, location of the sinus node (SN) and phrenic nerve (PN), and their spatial relationship to the ablation line (AbL) were assessed.

**Results:**

CB lines were present in 98% of patients, with gaps identified in all SVC segments, most frequently posterior (73%), with a median gap width of 23.9 (14.3–37.8) mm. All gaps expressed decremental properties in the EP study. Ablation was performed in 35 (42%) patients and was achieved in all cases using targeted segmental ablation, closing the gaps between the CB lines.Median ablation duration was 88 (66; 161) seconds with a median of 8 (6–12) radiofrequency applications. SN was separated from SVC with CB line in 98% of cases, what excluded necessity to ablate in this area, distance from the AbL to the SN exit zone was 8.9 (6-14.7) mm. PN was located outside the areas of preferential conduction in all cases, distance from AbL to the PN was 10.4 (6.7–12.1) mm. No complications occurred.

**Conclusions:**

HD mapping-guided, targeted segmental SVCI using RF energy is feasible and safe.

**Graphical Abstract:**

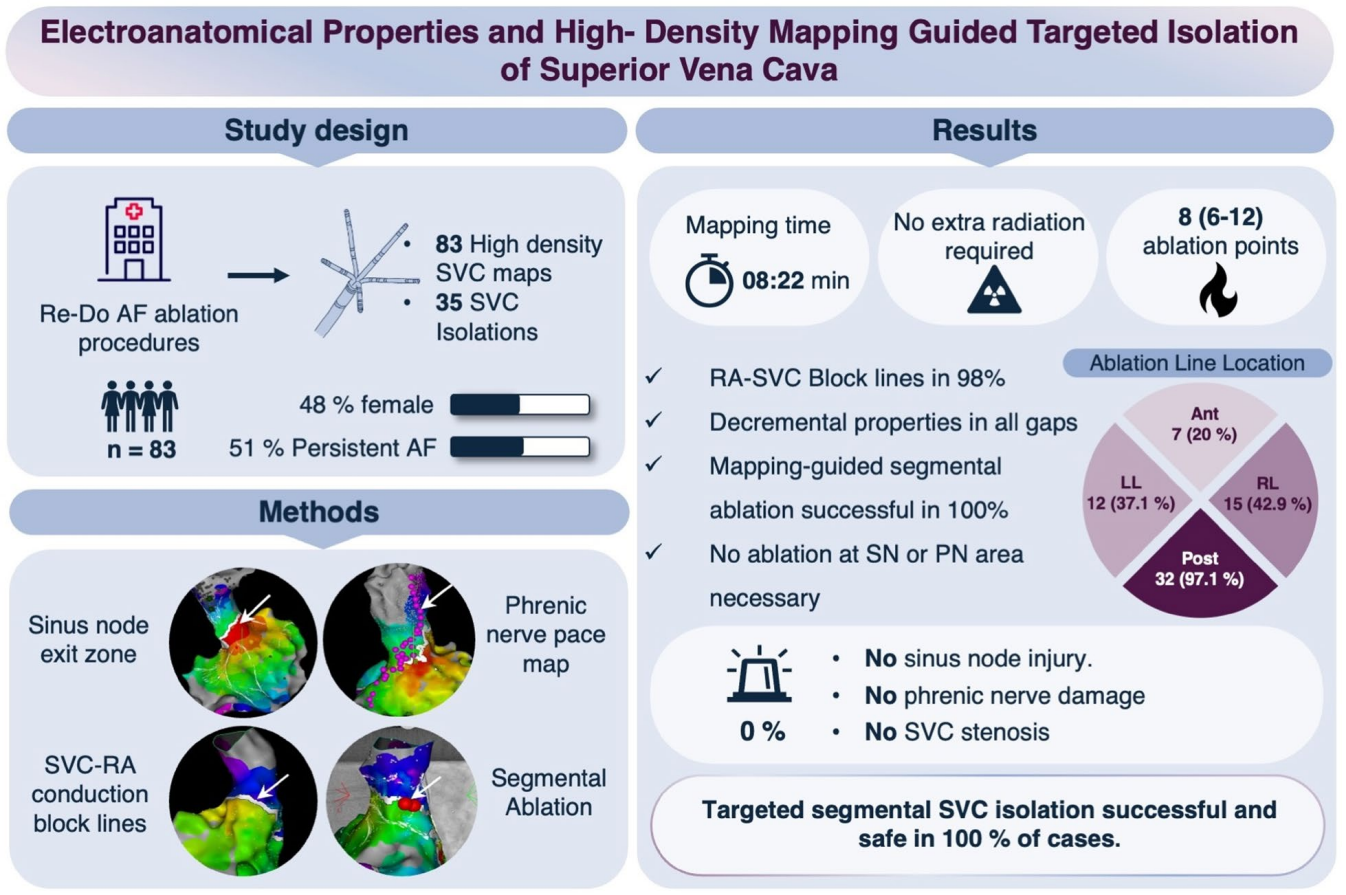

## Key learning points

- In 98% of patients, the superior vena cava (SVC) was separated from the right atrium (RA) by conduction block (CB) lines containing gaps that represented preferential RA-SVC conduction. This allowed targeted segmental SVC isolation and minimized the extent of ablation.
- Gap distribution was non-uniform; all gaps demonstrated decremental conduction properties.
- In all patients requiring SVC isolation, sinus node was separated from SVC by CB line, eliminating the need of ablation at this area.
- Pace-mapping of the phrenic nerve (PN) showed that its course was located outside the areas of preferential conduction, therefore ablation at PN capture sites was not required.
- High-density mapping-guided SVC isolation is feasible and safe and should be considered the preferred approach when using radiofrequency energy.

## Introduction

The superior vena cava (SVC) is one of the most important sources of non-pulmonary vein (non-PV) triggers of atrial fibrillation (AF), accounting for 25–40% [1–3]. SVC myocardial sleeves, due to their heterogeneity and presence of arrhythmogenic embryonic sinus venosus tissue, are capable of spontaneous firing and, consequently, initiation of AF [4, 5]. SVC isolation (SVCI) plays a significant role in treatment of AF and, when performed additionally to pulmonary vein isolation (PVI) may increase the success rate of the procedure [6, 7]. However, the vicinity of the phrenic nerve (PN) and sinus node (SN) poses the potential risk of complications and circumferential ablation can lead to SVC stenosis [8]. Segmental SVCI targeting the preferential conduction on the posterior right atrial (RA) wall has been shown to be feasible and effective [9]. Other studies have shown that RA-SVC connection may be located beyond the posterior segment, which makes high-density (HD) mapping essential for visualizing conduction gaps and minimizing RF applications [10–12]. Still, data from high-density (HD) mapping of the SVC remain limited. In the present study we sought to assess the electroanatomical properties of the SVC, define the spatial relationship with adjacent structures such as PN and SN, and assess the feasibility, efficacy, and safety of HD mapping-guided segmental SVCI in patients with recurrent AF.

## Methods

### Study population

This prospective descriptive cohort study included consecutive patients who underwent HD 3D mapping of RA and SVC, along with SVCI in selected cases, between 2022 and 2025 as a part of repeat ablation procedures for AF. HD 3D mapping was conducted using the CARTO mapping system (Biosense Webster Inc., Diamond Bar, CA, USA) and a multipolar mapping catheter (Pentaray, Biosense Webster). All patients provided informed written consent. The study was approved by the local ethics committee and conducted in accordance with the Declaration of Helsinki.

### Mapping protocol

All antiarrhythmic drugs were discontinued at least 5 half-lives prior to the procedure. Procedure was performed under deep sedation with propofol and fentanyl. As the first step of the repeat ablation for AF, dual transseptal puncture was performed, followed by mapping and ablation in the left atrium (LA). Re-PVI and, if indicated, substrate modification was performed in presence of PV reconnections or low voltage areas in the LA, respectively.

After completing LA ablation, both the mapping and ablation catheters were withdrawn in the RA. An HD 3D Map of the SVC and the superior portion of the RA, including the SN area, was created in sinus rhythm (SR). Mapping points were acquired automatically, based on filter settings such as cycle length (CL), usually set to 800–1200 ms, local activation time (LAT), position stability and respiratory gating. LAT for each point was calculated using wavefront algorithm. After completion of the map, a contact force-sensing ablation catheter (Thermocool SmartTouch Surround Flow or QDOT, Biosense Webster) was positioned in the SVC. High-output pacing (bipolar,10 mA at 2ms pulse width, contact force 5–15 g) was performed to identify capture of the PN in SVC and RA. Areas with PN capture were annotated on the map using location-only points.

**Fig. 1:**
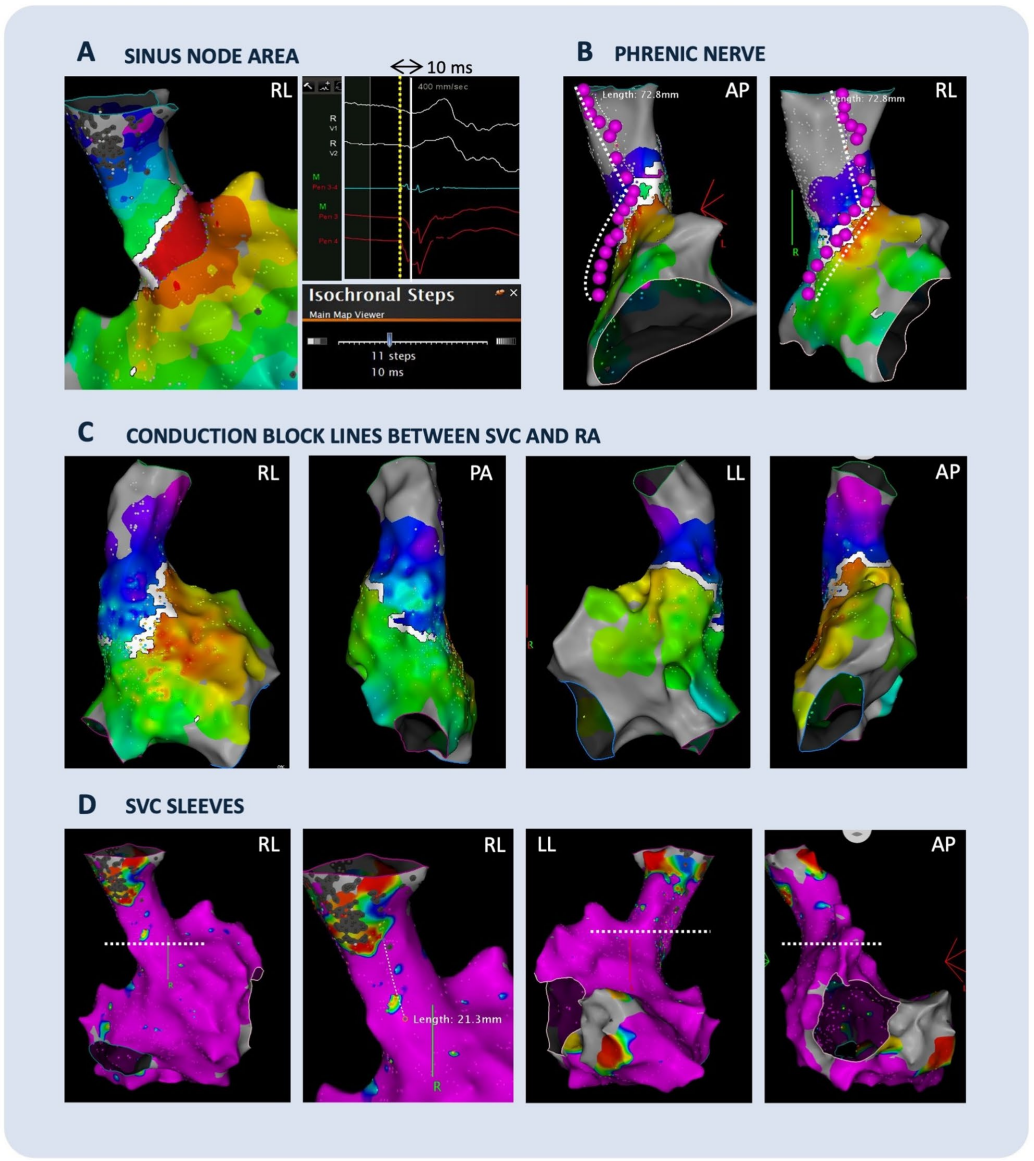
Measurements performed in the study. **A** Sinus node EZ measurement: on the left, isochronal map of the RA with 10 first ms of CL visualized in red; on the upper right, EGM of the earliest signal, annotated 10 ms before the onset of the P wave in 12 lead ECG; on the lower right: corresponding steps of the isochronal map in ms. **B** Pace map of the PN (purple dots) and measurement of PN length (white dotted line). **C** Conduction block lines (white lines) between the SVC and RA in activation maps (**D**) Measurement of the length of electrically active SVC sleeves (white dotted line: SVC-RA junction). AP, anteroposterior; EGM, electrogram; EZ, exit zone; LL, left lateral; PA, posteroanterior; PN, phrenic nerve; RA, right atrium; RL, right lateral; SVC, superior vena cava

All maps and electrograms were stored and carefully analyzed after the procedure. Only points acquired during SR were kept, points acquired during extrasystoles were deleted. The SVC was divided into 4 segments (Fig. 4A): anterior, right lateral (RL), posterior, left lateral (LL). The following mapping characteristics were assessed and the following measurements performed:

1. SN exit zone (EZ): defined as the area of the earliest 10 ms activation time on the endocardial isochronal map, (cm^2^), Fig. 1A
2. PN course: location and length of PN line, Fig. 1B
3. Conduction block (CB) lines: presence, location and length of CB lines between the SVC and RA as well as presence and location of gaps in CB lines was assessed. CB lines were visualized by the Carto “Early Meets Late” algorithm, depicted as white lines, and defined by LAT-difference > 20% of mapped CL of 2 adjacent points (Fig. 1C).
4. Electrically active SVC sleeves: length of the electrically active SVC sleeves was measured in four segments (Fig. 1D). Sleeves were measured from the anatomical SVC-RA junction to the highest mapping point in the SVC with voltage > 0.5mV in SR.

### EP study

**Fig. 2:**
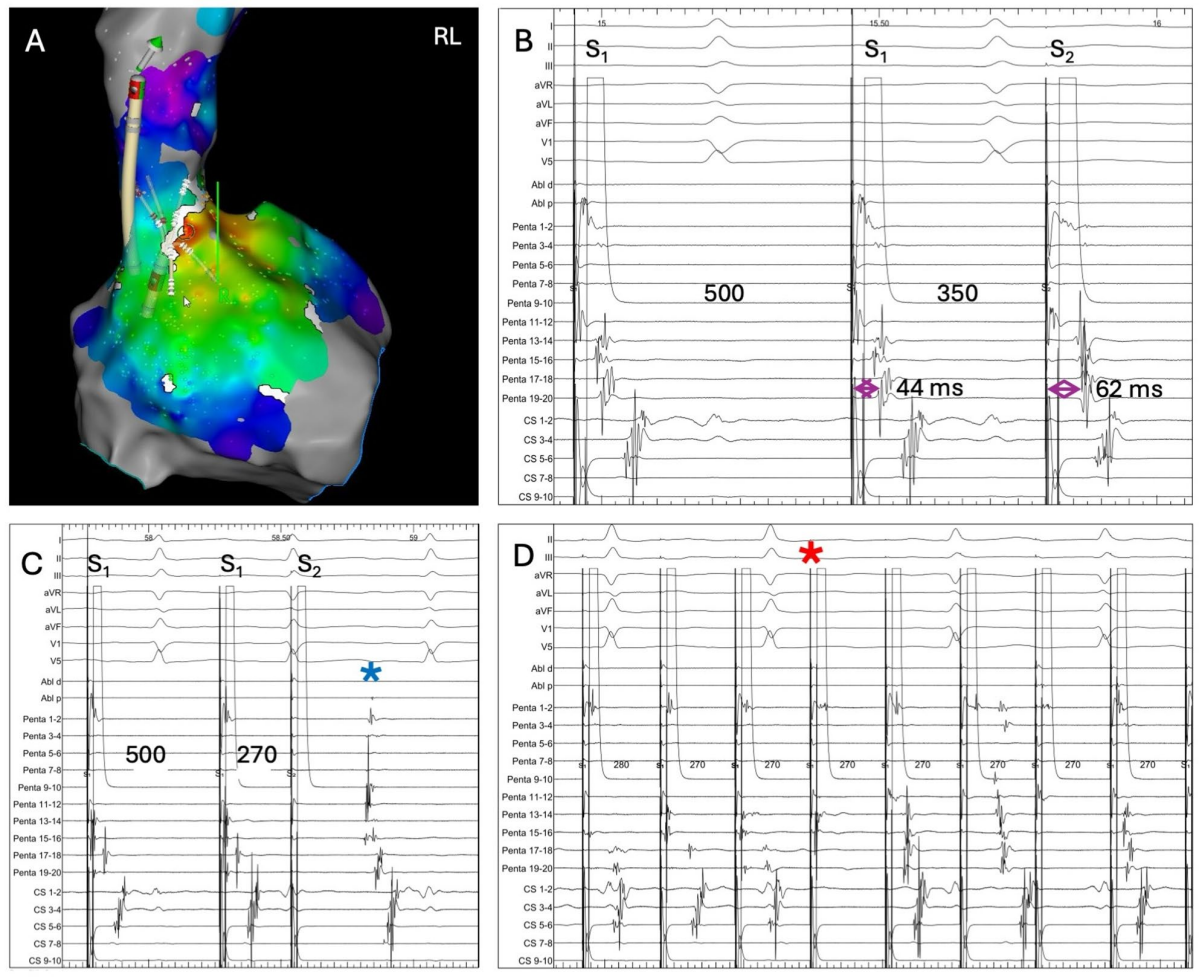
Electrophysiological study of the SVC. **A** Position of the multipolar catheter with some splines 1-2, 3-4, 5-6, 7-8, 9-10, and 11-12 located above and splines 13-14, 15-16, 17-18, and 19-20 located below the block line. **B** Programmed stimulation from SVC splines 7-8, demonstrating decremental conduction from the SVC to the RA; speed 200 mm/s (**C**) ERP of the SVC: 270 ms (blue asterisk- sinus beat); speed 100 mm/s (**D**) Wenckebach of the SVC at CL 270 ms (red asterisk), speed 100 mm/s. CL, cycle length; ERP, effective refractory period; RA, right atrium; RL, right lateral projection; SVC, superior vena cava

Electrophysiological study of the SVC was performed (Fig. 2). A Pentaray catheter was positioned along the CB line, with some splines located above and others below it. Stimulation was delivered from a spline located above the line, within the SVC. Pacing was performed with a train of five stimuli at a basic CL of 500ms, followed by S2 starting at 400ms and shortened in 20 ms decrements. Conduction to the RA was assessed. Effective refractory period (ERP) and Wenckebach CL of the SVC were determined.

### SVC ablation

**Fig. 3:**
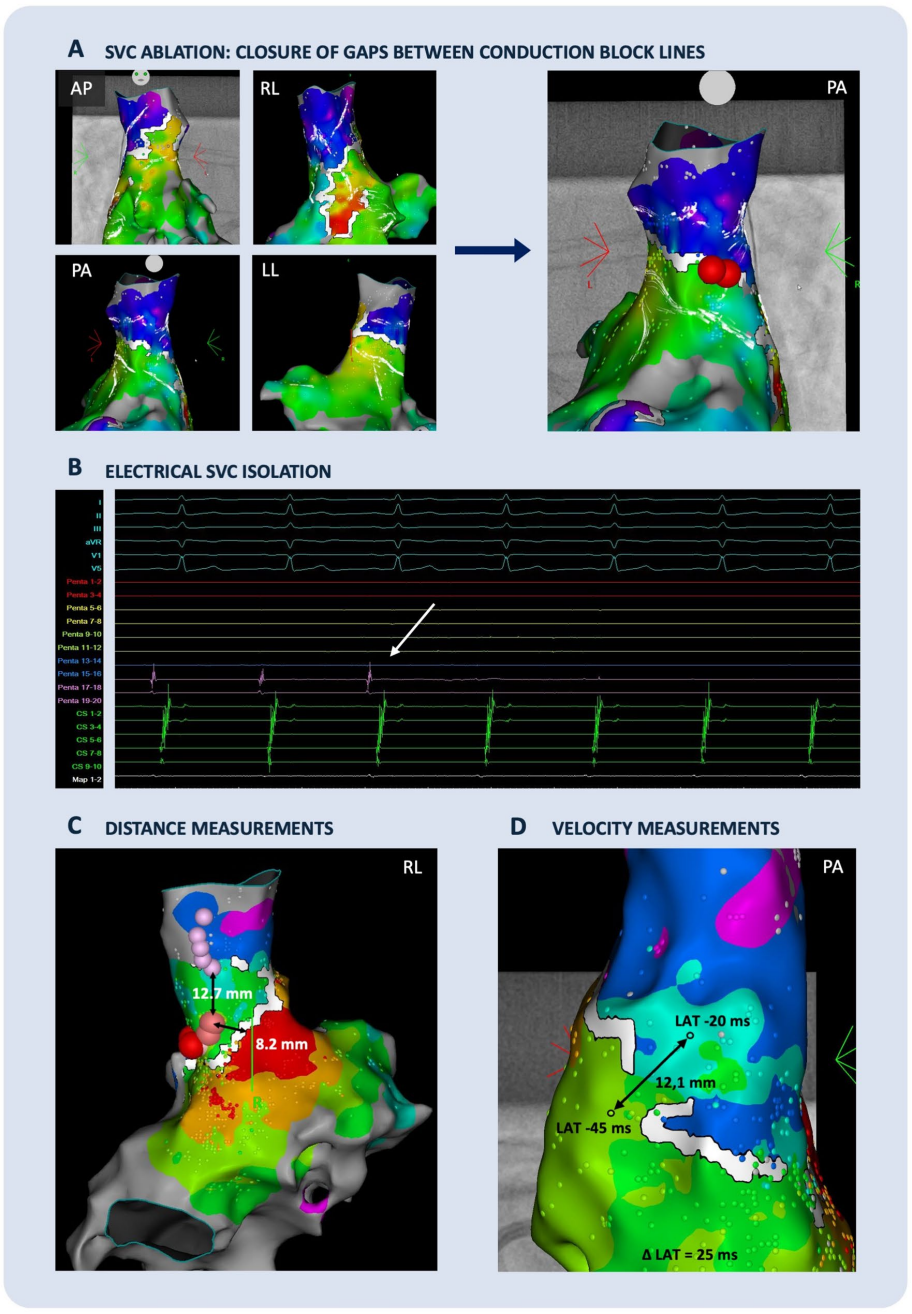
**A** Ablation of the SVC with closure of gaps between conduction block lines (red dots indicate ablation points). **B** Moment of electrical isolation of the SVC (white arrow). **C** Distances from the ablation lines to PN and SN. **D** Conduction velocity across the gap in the block line. AP, anteroposterior; LL, left lateral; PA, posteroanterior; PN, phrenic nerve; RL, right lateral; SN, sinus node; SVC, superior vena cava

SVC ablation was performed either in presence of non-PV triggers originating from the SVC (spontaneous or high-dose isoproterenol induced), empirically, in cases with isolated PVs and absence of substrate in LA. The procedure was carried out in SR, with radiofrequency (RF) energy, point-by-point, at power of 40–50 W, using an irrigated tip 4 mm ablation catheter with contact force sensing (Thermocool Smart Touch or QDot, Biosense Webster), targeting the ablation index of 400, with an interlesion distance of < 6 mm according to CLOSE protocol. In all cases an electrophysiological, rather than anatomical approach was applied: the ablation was targeted by closing the gaps between the CB lines (Fig. 3A). SVCI was confirmed when no signals were recorded on the multipolar mapping above the SVC-RA junction during SR (Fig. 3B). Post-procedure, ablation characteristics including total length of the ablation line, number of ablation points, distance to PN and SN and conduction velocity across the gaps were assessed (Fig. 3C and D).

### Statistical analysis

Continuous variables were assessed for normality and, due to their non-normal distribution, are presented as median and interquartile range (25–75%). Categorical variables were presented as percentages. As the study was descriptive in nature, no inferential statistical analyses or hypothesis-driven group comparisons were performed. Statistical analysis were performed using Microsoft Excel (Microsoft corporation, Redmond, WA, USA).

## Results

### Study population

A total of 83 Patients were included in the study: 40 (48%) were female; median age was 70 years (IQR 63–75), and 42 (51%) had persistent AF. Most patients received antiarrhythmic medication prior to ablation: 73 (88%) betablocker, 5 (6%) amiodarone, and 5 (6%) flecainide. Four (4.8%) patients had heart failure with an ejection fraction < 35%. Baseline patient characteristics are shown in Table 1.

**Table 1:** Patient baseline characteristics

Baseline characteristics	Total (*n* = 83)
Age (years)	70 (62.5; 75)
Female gender	40 (48.2%)
Persistent AF	42 (50.6%)
CHA2DS2-VA-Score	2 (1; 4)
Body mass index, kg/m^2^	27.1 (23.7; 29.3)
Hypertension	55 (66.3%)
Diabetes Melitus	18 (21.7%)
Coronary artery disease	29 (34.9%)
Dilative cardiomyopathy	9 (10.8%)
LVEF < 35%	4 (4.8%)
*Preprocedural antiarrhythmic medication*	
Betablocker	73 (88%)
Amiodarone	5 (6%)
Flecainid	5 (6%)

*AF *atrial fibrillation, *LVEF *left ventricular ejection fraction

### Electrophysiological study

In all cases we could see the decremental SVC properties with prolongation of the conduction time to the RA by decreasing the S2 coupling interval. Median ERP was 295 (IQR 226–352) ms, Wenckebach CL was 334 (IQR 282–403) ms. There were no cases of inadvertent AF induction during the EP Study. In two cases, episodic high-frequency fibrillatory activity, originating from SVC with conduction to the RA, initiated AF. After SVC isolation the high frequency activity persisted in SVC, while SR was maintained in RA. In one case sustained focal atrial tachycardia, originating from the upper part of crista terminalis was induced and successful ablation was performed.

### Mapping and ablation characteristics

High density 3D maps were obtained in all 83 patients with 753 (IQR 527–1114) mapping points and total map acquisition time of 8.2 (IQR 6.2-11-3) min. No cases of silent SVC were observed. Mapping and ablation characteristics are summarized in Table 2. Neither fluoroscopy nor contrast dye admission was required.

**Table 2:** Mapping and ablation characteristics

Mapping characteristics	Total (*n* = 83)			
Number of points	753 (528–1115)			
Mapping time (mm: ss)	08:22 (06:16 − 11:27)			
PN capture	52 (62.7%)			
PN length (mm)	21.9 (15.6–30.4)			
PN location (segment)	RL		Post	
	51 (98.1%)		2 (3.9%)	
SN-EZ area (cm²)	1.2 (0.8; 2.6)			
SN-EZ location (segment)	RL	Ant	LL	Post
	71 (85.5%)	35 (42.2%)	11 (13.3%)	14 (16.9%)
Presence of CB lines	81 (97.6%)			
Number of CB lines	2 (1; 2)			
CB line location (segment)	RL	Ant	LL	Post
	78 (96.3%)	64 (79%)	56 (69.1%)	40 (49.4%)
Presence of gaps	81 (97.6%)			
Number of gaps	1 (1; 2)			
Number of gaps in map	1 gap	2 gaps	3 gaps	4 gaps
	47 (58%)	27 (33.3%)	6 (7.4%)	1 (1.2%)
Location of gaps (segment)	RL	Ant	LL	Post
	26 (32.1%)	25 (30.9%)	32 (39.5%)	59 (72.8%)
Gap width (mm)	23.9 (14.3; 37.8)			
Velocity in gap (m/s)	0.62 (0.4; 0.9)			
SVC sleeve circumference (mm)	76.5 (69.8; 85.8)			
SVC sleeve length (mm)	RL	Ant	LL	Post
	22.6 (14.5; 28.6)	24.7 (19.9; 33.3)	24 (17; 28.5)	22.3 (16; 27.6)

**Ablation characteristics**	Total = 35			
SVCI achieved	35/35 (100%)			
Length of ablation line, mm	28.3 (19.3; 39.8)			
Number of RF applications	8 (6; 12)			
Ablation duration (seconds)	88 (66; 161)			
Distance ablation line – PN, mm	26/35, 10.4 (6.7; 12.1)			
Distance ablation line - SN-EZ, mm	8.9 (6; 14.7)			
Overlap ablation line - SN-EZ	0 (0%)			
Overlap ablation line - PN	0 (0%)			
Circular ablation line	0 (0%)			
Ablation line location (segment)	RL	Ant	LL	Post
	15 (42.9%)	7 (20%)	12 (37.1%)	32 (97.1%)
Distance ablation line - SVC sleeve end, mm	22.5 (16.2; 29.1)			

*Ant *anterior, *CB *conduction block, *LL *left lateral, *PN *phrenic nerve, *Post *Posterior, *RL *Right lateral, *SVC *superior vena cava, *SVCI *Superior vena cava isolation, *SN-EZ *Sinus Node Exit Zone

#### Sinus node exit zone

**Fig. 4:**
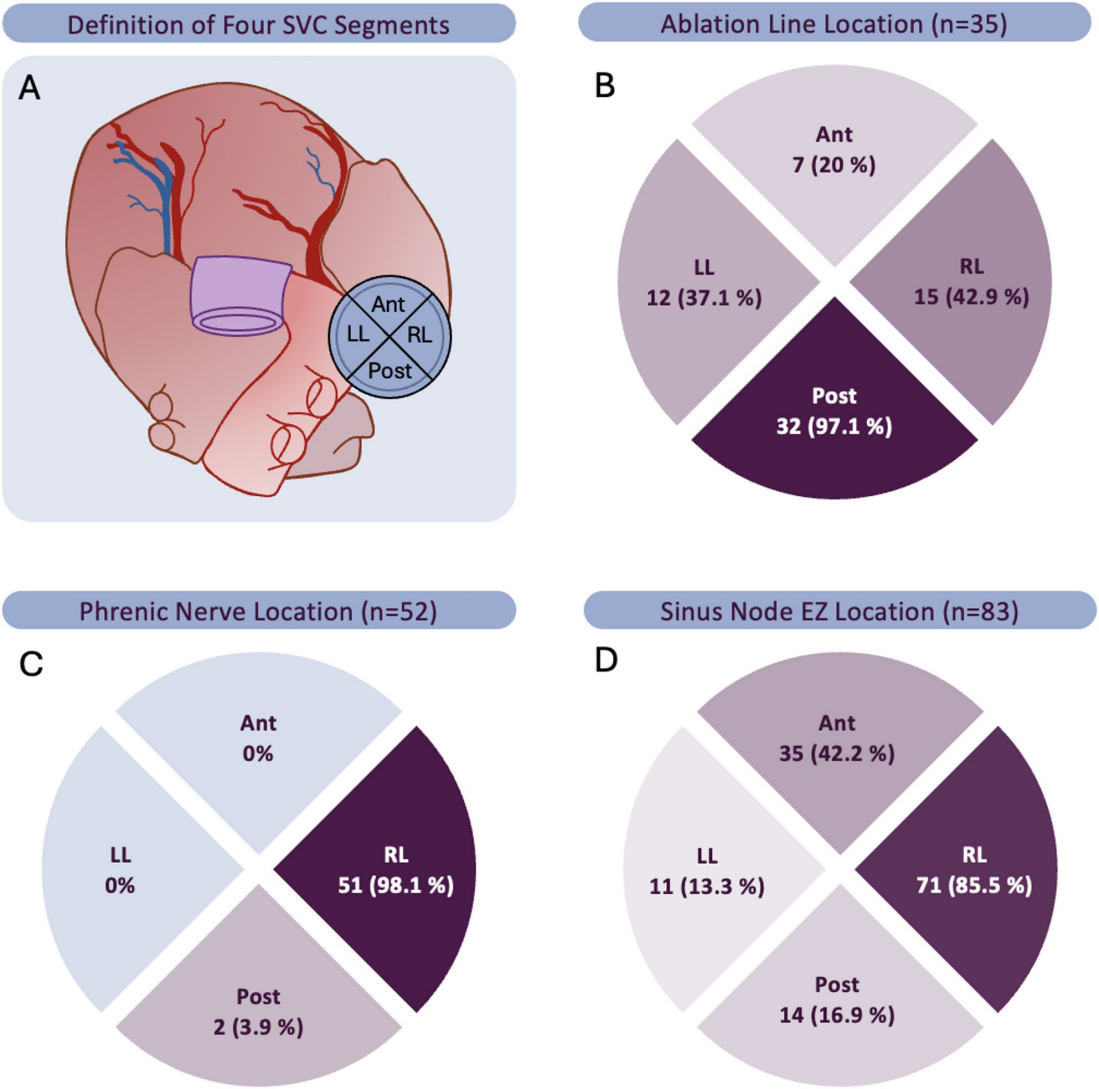
**A** Definition of four SVC segments in our study; (**B**) Location of the ablation line; (**C**) Location of PN with pace map; (**D**) Location of the SN-EZ (first 10 ms of activation). Ant, anterior; LL, left lateral; Post, posterior; RL, right lateral; SVC, superior vena cava, SN-EZ, sinus node exit zone

The SN EZ was 1.2 (IQR 0.8–2.6) cm^2^ and was separated from the SVC by a CB line in all maps except two cases. It was located predominantly in the RL segment in 71 (85.5%) of patients, less frequently in anterior *n* = 35 (42.2%), posterior *n* = 14 (16.9%) and LL *n* = 11 (13.3%) segments (Fig. 4D), if the SN EZ was located in more than one segment in a patient, all corresponding segments were documented. The distance from the ablation line (ABL) to the outer border of the SN EZ was 8.9 (IQR 6-14.7) mm. There were no instances of the ablation line crossing the SN EZ or sinus rhythm acceleration during ablation prompting the ablation discontinuation, or any evidence of SN injury after the procedure.

#### Phrenic nerve

PN capture in the lower part of the SVC could be achieved in 52 (62.7%) of patients, with captured PN length of 21.9 (IQR15.6-30.4) mm and dominant PN location in the RL segment in 51 (98%) of patients, in 2 (3.9%) of patients PN could be captured in posterior segment (Fig. 4C). The distance from PN to the ablation line was 10.4 (IQR 6.7–12.1) mm. No RF applications were performed at sites with PN capture as its course was located outside the areas of preferential conduction. No cases of PN paresis (incl. transient injury) were observed.

#### SVC sleeves

**Fig. 5:**
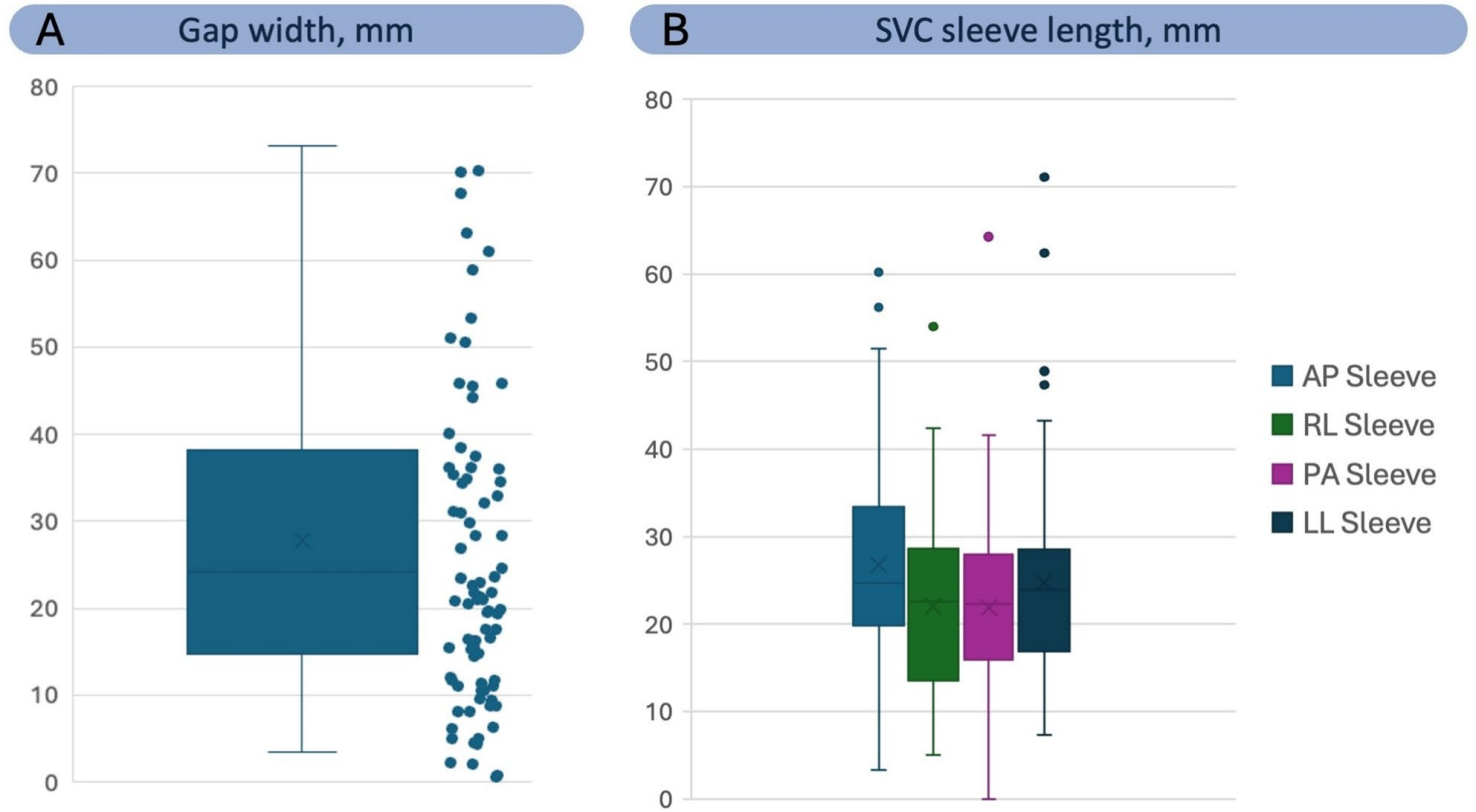
**A** Median gap width and (**B**) median SVC sleeve length in each of four segments in the study cohort. Ant, anterior; LL, left lateral; Post, posterior; RL, right lateral; SVC, superior vena cava

The length of electrically active SVC sleeves was asymmetric, with the longest median length anterior 24.7 (IQR 19.9–33.3) mm, LL 24 (17-28.5) mm, RL 22.6 (IQR 14.5–28.6), and the shortest posterior 22.3 (IQR 16-27.6) mm (Fig. 5B).

#### Conduction block lines

In 81cases (97.6%) CB lines between the SVC and RA were identified, as well as areas of preferential conduction between them with a median width of 23.9 (14.3–37.8) mm (Fig. 5A) and a median velocity of 0.6 (0.4–0.9) m/s. Of two patients without CB lines, one was 32-year-old female with paroxysmal AF, and one 66-year-old male with persistent AF. Most patients (*n* = 47, 58%) had one gap, 27 (33.3%) two gaps, 6 (7.4%) three gaps and one patient had 4 gaps. Gaps could be identified in all segments, most frequently in the posterior segment (*n* = 59, 72.8%), less often in LL (*n* = 32, 39.5%), anterior (*n* = 25, 30.9%) and RL (*n* = 26, 32.1%) segments.

#### Ablation

In 35 (42.1%) patients SVCI was performed, in 10 (29%) cases due to non-PV triggers (in 3 patients spontaneous, in 7 induced with isoproterenol), and in other cases empirical: as a part of redo procedure in patients without low voltage substrate. SVCI could be successfully achieved in all 35 patients. Decision where to perform ablation was solely based on the identified gaps within the CB lines. Most frequently ablation was performed posteriorly (*n* = 32, 97.1%), less often in RL (*n* = 15, 42.9%), LL (*n* = 12, 37.1%) and anterior (*n* = 7, 20%) segments (Fig. 4B). The mean length of the ablation line was 28.3 (IQR 19.3–39.8) mm, with 8 (6–12) applications and total RF energy delivery duration of 99 (66–161) seconds. The SVC isolation could be achieved with 2.1 (1.7-3.0) ablation points. Due to the presence of CB lines, there were no cases, where circumferential SVC ablation was necessary. The ablation line covered less than half of the SVC circumference (41.1%, IQR 26.5–56.2).

## Discussion

The present study demonstrates findings of HD 3D mapping of the SVC and RA and describes mapping-guided targeted segmental SVCI approach. These are the main findings:

- In 98% of patients, the SVC was separated from the RA by conduction block (CB) lines containing gaps that represented preferential RA-SVC conduction. This allowed targeted segmental SVC isolation and minimized the extent of ablation.
- Gap distribution was non-uniform; all gaps demonstrated decremental conduction properties.
- In all patients requiring SVCI SN was separated from SVC by CB line, eliminating the need of ablation at this area.
- Pace-mapping of the PN showed that its course was located outside the areas of preferential conduction, therefore ablation at PN capture sites was not required.
- HD mapping-guided SVC isolation is feasible and safe and should be considered the preferred approach when using RF energy.

Three approaches to SVCI have been described in the literature: (1) anatomically based circumferential ablation, (2) segmental ablation without use of HD activation mapping, (3) electrophysiologic approach guided by HD 3D Mapping.

1. The anatomy-based circumferential approach to SVCI involves ablation at the SVC-RA junction, typically defined by angiography or intracardiac echocardiography (ICE) [13–15]. Its efficacy and safety are limited by the close proximity of SN and PN, resulting in a considerable rate of incomplete isolation in up to 18% of patients [2, 7]. Moreover, the wider extent of ablation carries a potential risk of late SVC stenosis or SVC syndrome [8, 16]. Anatomical studies have further demonstrated that myocardial extensions from RA into SVC are less likely to be circumferential, indicating that complete circumferential SVCI may not be necessary [17].
2. Segmental SVC isolation has been proposed as an effective alternative to the circumferential anatomical approach. Gianni et al. described a novel technique, based on embryological data, suggesting preferential conduction from the RA into SVC, predominantly through the posterior wall [9, 18]. In their study, HD activation map was not performed. Instead, irrespective of the site of breakthrough, ablation was started at the septal aspect of the SVC-RA junction in all patients, followed by RA posterior wall, until the isolation was achieved. This approach was effective in 98% of patients, with symptomatic PN paresis developed in 2% of cases. Chen et al. reported a C-shaped SVC ablation line in 12 patients, sparing the lateral SVC segment [19]. Although an SVC map was obtained, it was used solely to identify the SN location rather than to assess the conduction pattern. Given the heterogenous distribution of myocardial sleeves, the omission of RA-SVC activation mapping represents a limitation.
3. Electrophysiological approach of SVCI using the HD 3D mapping is an emerging strategy, as it allows to access the individual SVC-RA conduction pattern and thereby facilitates the segmental targeted ablation. Recent studies have demonstrated the presence of conduction block (CB) between the RA and SVC in patients with AF; however, the reported prevalence varies widely ranging from 8.8 to 100% [11, 12, 20–22]. This variability likely results from differences in representation of CB lines, the mapping system used (CARTO vs. RHYTHMIA), and the mapping parameters applied for block line visualisation.

Two studies used the Rhythmia system and isolate SVC based on the results. Tanaka et al. analysed 113 maps from patients with AF, observing RA-SVC conduction block in about half [22]. These were classified into three shapes: type I (straight), type J (reverse J) and type U (reverse U) with a mean length of 18.8 mm. Ablation was performed by connecting two ends of the CB line, whereas circumferential ablation was applied in patients without CB lines. Importantly, the presence of identifiable CB lines was associated with fewer RF deliveries for SVCI and lower risk of complications. Yamashita et al. did not describe the conduction block lines themselves but instead reported a mean of 2.5 of breakthroughs from RA to SVC per patient along with spiral activation of the SVC. Ablation was performed selectively at these breakthrough sites, achieving a 100% success rate without complications [20].

Two further studies evaluated SVC isolation with CARTO mapping system, using the extended early-meets-late tool for visualization of the CB lines. Inagaki et al. reported the presence of CB lines in 81.5% of patients with AF, and patients in CB lines group required fewer RF applications for SVCI [11]. For visualization of lines “low threshold” was initially set individually and, if SVCI was not achieved after gap closure, increased stepwise by 5%. Matsunaga et al. reported presence of block lines in all patients with AF; however, the criteria of the CB lines visualization were set up individually for each patient12. Interestingly, in HD RA-SVC maps in patients without known AF, no CB lines have been reported [21, 23].

Our study, performed with the CARTO system, showed presence of CB lines in 98% of patients. Mapping acquisition time and number of mapping points were comparable with the previous data. For visualisation of block lines, a fixed “Low Threshold” of the early-meets-late algorithm of 20% was applied in all patients. Successful segmental isolation was achieved by targeting the gaps between the block lines with the median ablation line length involving less than half of the SVC circumference, and without any case requiring circumferential isolation. As the location and width of gaps, as well as the location of SN and PN varied among patients, we believe that (1) HD mapping is essential for visualisation of RA-SVC conduction and adjacent structures as SN and PN (2) in patients with CB lines, RF delivery should be limited to preferential conduction sites through the gaps, rather than applying anatomically guided ablation. We did not encounter any of the mapping limitations reported in previous studies, such as AF induction, absence of SVC signals or junctional rhythm instead of SR [12]. However, it is noteworthy, however, to mention, that correct adjustment of mapping filters is essential to enable rapid map acquisition with accurate point annotation. In particular, the correct CL (usually set to 800–1200 ms) filter as well as application of IC Pattern matching is crucial, as it prevents acquisition of mechanically induced supraventricular extrasystoles (SVES), which are frequently observed, mapping the upper RA and SVC. In our study SVCI was achieved with a median of two ablation points, consistent with initial report on SVCI [24]. However, the total number of ablation points was higher in our cohort, likely due to use of 3D HD mapping, which enabled more precise visualization of the conduction gaps, compared with the earlier study conducted before the era of 3D mapping.

### Electrophysiological properties

The data on electrophysiological properties of the SVC are limited. Decremental conduction, demonstrated with the extrastimuli delivering in both directions (from the SVC to RA and vice versa), has been previously reported [25]. In addition, Ishikura et al. described clearer visualisation of the electrical RA-SVC junction when pacing with the extrastimuli from the coronary sinus [10]. In our cohort EP Study with extrastimulus was performed to (1) confirm distinct RA-SVC conduction through a narrow area (2) induce high frequency activity in SVC. Decremental conduction from the SVC to RA was observed in all cases.

### Sinus node exit zone

The SN is located in the RA, at the junction with the SVC; accordingly, SN injury has been reported as a possible complication of SVCI [26, 27]. Since antral ablation of the SVC carries this risk, it was recommended to deliver the RF applications slightly above the anatomical RA junction [28]. Still, defining the SVC-RA junction might be challenging and the location of the SN EZ has also been reported to vary widely [12, 20, 29]. In line with these data, in our cohort it was most frequently observed in RL (85,5%) and anterior (42.2%) segments, although in some cases it was also present left lateral and posterior. Notably, none of the studies, using HD 3D mapping for SVCI, reported symptomatic sick sinus syndrome after ablation. These findings highlight the importance of performing activation mapping with direct visualization of SN, which allows to avoid the ablation in the SN area. Our study showed the presence of CB line between the SN and SVC in all cases. When the strategy of the SVCI by connecting the ents of CB lines is applied, the ablation at SN is excluded.

### Phrenic nerve

Since the average thickness of the SVC sleeve is approximately 1 mm, there is a relevant risk of PN damage during SVCI [30]. Symptomatic PN injury has been reported in about 2% of cases, associated with ablation at the posterolateral segment [9]. In our study the PN was predominantly located in the RL segment (98%), and in 2 cases posterior. In studies not employing HD mapping, a frequent inability to achieve complete SVC-RA isolation (13–18%) was reported due to PN capture at the potential ablation site [2, 7]. In contrast, studies, using the mapping-guided SVCI did not report this issue and generally achieved SVCI in all cases. Although some authors performed ablation on the PN capture sites with reduced energy (15–20 W) [11, 12], in our study such ablation was not necessary as the course of PN was outside the preferential RA-SVC conduction in all cases and the median PN-to-ablation line distance exceeded 1 cm.

### Alternative ablation and imaging techniques

Alternatively, to RF ablation, SVCI can be performed using other ablation techniques. Several studies have reported SVCI with cryoballoon, showing a suboptimal success rate of 81–94% and complications such as transient SN and PN injury in 8–19% and 19–21% of cases, respectively [31, 32]. Pulsed-field ablation, owing to its tissue specificity, is a promising technique for SVCI, with a reported 100% acute success rate, a varying risk of reversible complications (transient SN dysfunction 0.003-4.7% and transient PN stunning 0.004-64%), and no irreversible collateral damage has been reported [33, 34]. However, the use of both techniques is limited for first-time AF ablation procedures, and no data are available regarding the durability of isolation.

Some authors use intracardiac echocardiography (ICE) as an additional imaging tool to define the SVC-RA junction and to evaluate catheter position, particularly when PFA without 3D mapping is performed [2, 7, 33]. In our study, ICE was not required, as both the SVC-RA junction and catheter position were adequately visualized with 3D mapping.

### Limitations

This was a non-randomized, single-center observational study. All procedures were performed with a single mapping system. No follow up was conducted regarding AF recurrence or the long-term durability of SVCI, so the clinical effectivity remains unproven. The long-term benefit of SVC isolation using this selective approach should be studied in prospective randomized setting.

## Conclusion

RA-SVC connection is not uniform with regions of preferential conduction with decremental properties between fixed conduction block lines observed in 98% of the patients. HD 3D mapping guided segmental isolation of SVC by targeting preferential conduction is feasible and safe. Sinus node is separated from SVC by conduction block line in all patients which excludes necessity of ablation at this area. Pace-mapping of the PN showed that its course lays outside the areas of preferential conduction.

## Data Availability

No datasets were generated or analysed during the current study.

## Abbreviations

AF: Atrial Fibrillation
AbL: Ablation Line
Ant: Anterior
CB: Conduction Block
CL: Cycle Length
EAM: Electroanatomical Mapping
EGM: Electrogram
ERP: Effective Refractory Period
EZ: Exit Zone
HD: High-density
ICE: Intracardiac Echocardiography
LA: Left Atrium
LAT: Local Activation Time
LL: Left Lateral
LVEF: Left Ventricular Ejection Fraction
PN: Phrenic Nerve
Post: Posterior
PV: Pulmonary Vein
PVI: Pulmonary Vein Isolation
RA: Right Atrium
RF: Radiofrequency
RL: Right Lateral
SN: Sinus Node
SN: EZ-sinus node exit zone
SR: Sinus rhythm
SVC: Superior Vena Cava
SVCI: Superior Vena Cava Isolation
